# I know what I like, and I like what I know: Patient preferences and expectations when choosing an arts therapies group

**DOI:** 10.1016/j.aip.2021.101829

**Published:** 2021-09

**Authors:** Emma Millard, Jessica Cardona, Jane Fernandes, Stefan Priebe, Catherine Carr

**Affiliations:** a Unit for Social and Community Psychiatry, WHO Collaborating Centre for Mental Health Service Development, Queen Mary University of London, United Kingdom; b East London NHS Foundation Trust, United Kingdom

**Keywords:** Patient preferences, Psychiatry, Group arts therapies, Expectancy

## Abstract

Patient preferences deserve consideration as they play a role in engagement with psychosocial interventions. This study sought to understand more about preferences and expectations of the arts therapies, in order to support informed decision-making.

Semi-structured interviews were conducted with 17 participants in a trial of group music therapy, art therapy and dance-movement therapy. Audio recordings were transcribed. Data relating to the process of choosing their preferred arts therapies groups, expectations and experiences of the groups were analysed using framework analysis.

Three overarching themes were found relating to the experience of choosing an arts therapies group and subsequently attending it: past experiences of the art forms, social interactions in the groups and expectations of helpfulness. Familiarity and perceived capability were important factors for the therapy experiences.

Although each participant had a unique decision-making process, the themes offer understanding of common considerations when making a choice about engagement with the arts therapies. Clinicians should guide discussions around past experiences of the art forms, group dynamics and therapy aims during shared decision making for the arts therapies.

## Highlights

• Past experiences and capability in the art forms informed choices.
• Participants had mixed expectations of the social interactions in the groups.
• Participants considered the ways in which the arts therapies would be helpful for them.
• These aspects should be considered during collaborative decision-making for the arts therapies.

## Introduction

Collaborative decision-making and accommodation of preferences in mental healthcare is recommended by international guidelines (World Health Organisation, 2021) and is a crucial part of person-centred care (Gabrielsson et al., 2015). Working alongside patients to understand and respect their values is in line with recovery-oriented practice (Leamy et al., 2011), and is an ethical obligation for mental health practitioners (Norcross & Cooper, 2021).

Expectations about therapy are likely to be an important aspect of decision-making, as well as being relevant to the therapeutic process (Arnkoff et al., 2002). A recent meta-analysis found a significant association between outcome expectation and post-treatment outcomes in 81 studies of psychotherapy, i.e. expectations of the therapy being helpful was associated with better outcomes (Constantino, Visla, et al., 2018). The same research team also found that a positive perception of the treatment was associated with improved outcomes (Constantino, Coyne, et al., 2018). Wampold and Imel (2015) suggest that expectation-setting is one of three common mechanisms of change across psychotherapy approaches (Wampold & Imel, 2015). This is known as expectancy and specifically refers to the expected changes that patients will see in themselves when they attend therapy. Expectancy about helpfulness may be linked to the beliefs that patients hold about the causes of their illness (Khalsa et al., 2011; Sandell et al., 2011; Sidani et al., 2009), e.g. if they believe depression is caused by a chemical imbalance, they are likely to expect pharmacological interventions to be the most helpful. Seeking therapy in itself pre-empts positive change, as the act of engaging in therapy suggests that the patient expects to find it useful in reducing their distress; seeking an intervention may provide the patient with a sense that they are able to take action and have agency over their situation. Other researchers also suggest that expectancy and levels of hope are important contributors to the therapeutic process (Greenberg et al., 2006; Snyder et al., 1999).

The arts therapies are a group of psychosocial interventions including music therapy, art therapy, dramatherapy and dance-movement therapy. In mental health services in the UK, arts therapies may be provided by a team, covering a number of different arts modalities (Jones, 2020), which could provide opportunities for patients to choose their preferred arts therapies modality. The arts therapies share many features, and there is considerable overlap between the underlying approaches of each modality (Carr et al., 2020; Koch, 2017), however there are also clear differences in the art form being used (Crawford & Patterson, 2007; Fenner et al., 2017). Therefore patients’ expectations and preferences could play a key role in their engagement with the therapeutic process.

Arts therapies are often offered in a group format in UK mental health services (Jones, 2020). The benefits of group therapy are well-documented, with the aims being slightly different to individual therapy (McRoberts et al., 1998; Yalom & Leszcz, 2020). Groups are seen as a more realistic representation of the real world, and the networks of people that we engage with in our lives, than individual therapy (Foulkes & Pines, 1990). People can try out new ways of being with one another, potentially escaping some ‘stuckness’ in their personal relationships. In the arts therapies, there is another dimension to these relationships – the use of the art form. The art form can be used for non-verbal expression, which allows for communication of feelings and experiences that can be difficult to convey, make sense of or put into words (Jones, 2020). In groups, the art forms offer a way for group members to connect with each other and the therapist non-verbally (Carr et al., 2020; Davies et al., 2015).

The MATISSE study examined group art therapy compared to treatment as usual for people with schizophrenia (Crawford et al., 2012). The study did not find any difference in the primary outcome (reduced symptoms) between groups. However, a major problem in this study was high dropout levels; 39 % of people in the art therapy arm did not attend any sessions, and among those who did, attendance was very low. It was suggested that unclear expectations about art therapy from both patients and other healthcare professionals may have contributed to low attendance (Crawford et al., 2012). This is supported by other studies which have suggested that a lack of information about community psychotherapy groups is a key barrier to attendance (Dilgul et al., 2018), and that a mismatch in preferences is likely to be a significant contributor to dropout from psychosocial interventions (Windle, Tee, et al., 2019). Although preferences and expectations may not always be the same (Arnkoff et al., 2002), they are related, and can be constructed and elicited during shared decision-making processes.

Qualitative studies can offer an understanding of the expectations and preferences patients might bring to the process. In a study of patient experiences of group songwriting for long-term depression, participants reported that they did not know what to expect when starting the groups, which caused uncertainty and anxiety (Windle, Hickling, et al., 2019). Some had expectations of the groups which were not met, and they decided to stop attending. Other participants described their enjoyment of the intervention and the ways in which it had helped them to manage their symptoms and reintegrate in the community. Another study found that patients in a dance-movement therapy group took part in the group because they expected it to alleviate their symptoms, they expected to learn something new and also to receive peer support (Pylvänäinen & Lappalainen, 2018).

### The ERA trial

The ERA trial is a pragmatic randomised controlled trial to test the effectiveness of group arts therapies compared to an active control of group counselling for psychiatric patients in community mental health care. Participants had to be 18 years old or above, living in the community, with an ICD-10 diagnosis of F2 (schizophrenia and related psychotic disorders), F3 (mood disorders) or F4 (anxiety and other non-psychotic disorders) and at least moderate self-reported symptom level on the Brief Symptom Inventory (BSI) (Derogatis, 1983). Participants in the ERA trial were given a choice of arts therapies treatment modalities as part of the study design. Art therapy, dance-movement therapy or music therapy were offered, and participants were informed about these options by watching videos, which were developed by the study team in collaboration with patients and arts therapists. Dramatherapy was not offered for pragmatic reasons, i.e. study design and local resources. The decision aid videos involved footage of experiential sessions and interviews with patients about their experiences of the arts therapies. They lasted 2−3 min and there was one video per arts modality. Therapist interviews were not included in the videos so that the choice was clearly between arts modalities rather than the therapist’s characteristics or approach. Participants were then invited to choose the modality they would most want to engage in and were randomised to their preferred arts therapy group or group counselling.

Participants met their arts therapist for an initial meeting, which was structured and focused on introducing the participant to the aims of the group, the physical space where it was conducted, and to give them the opportunity to ask questions. The arts therapy groups were facilitated by an experienced arts therapist and a co-facilitator (qualified or trainee arts therapist) in accordance with a manual (Carr et al., 2020). The manual detailed 10 core principles and five core aims of group arts therapies. It was based mainly on humanistic, person-centred, resource-oriented with some scope for psychodynamic thinking. The groups all ran twice-weekly, 90-minute sessions, for 20 weeks. Due to higher numbers of people choosing art, two art therapy groups ran, one music therapy and one dance-movement therapy group. There were up to 10 participants in each group but in practice these numbers varied depending on availability and dropout. Assessment data was collected at baseline, post-intervention, and at 6 and 12 month follow up points by blinded researchers.

The trial design offered a unique opportunity to study the experiences of making a choice in the arts therapies, and the subsequent experiences of the groups. An increased understanding of the expectations that patients brought, both before and during their attendance of group arts therapies, is of relevance. This will aid comprehension of how preferences are formed, what factors play an important role for patients during decision-making, and how expectations shape the subsequent experiences once the therapy begins.

The following questions were designed to explore the decision-making process of patients who were part of the ERA trial for group arts therapies:

Research Questions:

• How did participants experience making a choice about their arts therapies treatment?
• How did participants’ preferences and expectations relate to their experiences of arts therapies?

## Methods

This study consisted of semi-structured interviews with patients who had taken part in the internal pilot stage of a randomised controlled trial of community arts therapies (ERA). It was given ethics approval as part of the ERA trial (REC Ref: 18/YH/0464) and the protocol was registered before the study commenced (Carr, 2018). This study was conducted and reported according to the COREQ guidelines for qualitative research (Tong et al., 2007).

### Participants

Participants were invited to take part in interviews if they had been part of the ERA internal pilot trial, had expressed an interest in taking part in interviews during their baseline assessment and had been randomised to the arts therapies intervention (n = 36). The sampling was conducted according to the ERA protocol, and stratified to include participants with high attendance (≥20 out of 40 sessions) and low attendance (<20 sessions), with representation of each diagnostic group (F2, F3 and F4), arts modality, gender, and a range of ages.

Initially it was planned to interview participants from all three study sites. However, due to the COVID-19 pandemic, the trial was paused after only the London site had completed the groups, therefore all interviewees in this study were London participants. They were interviewed between one and six months after the therapy groups ended.

### Design

The study involved semi-structured interviews with patients from the ERA trial. The topic guides were developed based on the Client Change Interview (Elliott et al., 2001) and adapted in collaboration with the ERA Lived Experience Advisory Panel (LEAP). They covered research questions relevant to this study, as well as questions about the participants’ general experiences of the groups for future analysis by the ERA team (see Appendix A). This was so that the participants would not need to undertake more than one interview, which could be time-consuming and emotionally demanding. The most relevant questions to this study were Q1-5 in the topic guide.

The interviews were planned to be conducted face-to-face, but due to the COVID-19 pandemic patients were invited to take part over Zoom video-conferencing or on the phone. The first author (EM) conducted all interviews, and JF attended one as an observer. Participants were sent £20 of supermarket vouchers in the post in recognition of the value of their time. Audio recordings were sent to an NHS-approved agency for transcription.

### Analysis

The data was analysed using a framework analysis approach (Rabiee, 2004; Richards, 2005) in NVivo 12 (QSR International Pty Ltd, 2018). After reading through for initial familiarisation, relevant information from five transcripts was coded by EM and JC to develop an initial coding framework. Through discussion with CC, it was decided that all transcripts needed to be involved in this initial coding, because of the wide range of experiences and characteristics of the participants. EM and JC then divided the remaining transcripts and coded these. Codes were discussed across a series of online meetings (EM and JC) and a virtual post-it note platform (mural.co) was used to enable live grouping of similar codes and naming of themes. The themes were used as columns in a framework matrix, with each participant as a different row. EM populated the framework with a summary of each participants’ experiences of the themes. These themes were then regrouped to look for further high-level themes. EM summarised the findings and the summaries were discussed amongst the study team. A draft of the manuscript was reviewed by a team of multi-disciplinary researchers.

### Study team

The background of the authors is important to consider as their life experiences will have had an influence on the study design and analysis (Berger, 2015). The first author (EM) is a music therapist and PhD researcher with three years of experience working in multi-modal arts therapies teams in NHS mental health services; she conducted all of the interviews and took the lead on the analysis and write-up. JC is a research assistant on the ERA trial and assisted with data-analysis and write-up. JF is a member of the LEAP who advised on the topic guide development, data collection and final write-up. SP is a clinical and academic psychiatrist and was involved in the study design and interpretation. CC is a music therapist and researcher, as well as the chief investigator of the ERA trial. She was involved in the current study design, analysis and write-up.

The variety of experiences in the study team brought different perspectives to the design, analysis and interpretation. Similarities between team members meant that the study was conducted through a specific lens: All team members are supportive of patient choice and autonomy and place a high value on the patient voice and experience. They are also members of the Unit for Social and Community Psychiatry, meaning that there was an emphasis on social relationships and connections throughout the data analysis and interpretation. The study was conducted with a pragmatic approach, looking for information which would be practically useful for clinical services (Shannon-Baker, 2016).

## Results

Overall, 17 participants agreed to take part in the interviews, from a potential pool of 36 (those who had been randomised to arts therapies groups in the London internal pilot). The mean age of participants was 46.4 (SD = 8.4) and the mean number of sessions attended was 18.6 out of a total of 40 (SD = 11.5). The mean time taken for the interviews was 40.6 min (SD = 17.3). Table 1 shows other characteristics of the interview participants.

**Table 1:** Participant characteristics.

Characteristic	Group	Number of participants	
		N	%
Past experience	Previous experience of arts therapies	13/17	76**%**
	Choice of arts therapies matched past experience	12/13	92**%**
Arts modality group	Art therapy	10/17	59**%**
	Dance-movement therapy	3/17	18**%**
	Music therapy	4/17	24**%**
Attendance	Did not attend	1/17	6**%**
	Low (<50 % of 40 sessions)	6/17	35**%**
	High (≥50 % of 40 sessions)	10/17	59**%**
Diagnosis	F20 - F29 Schizophrenia, schizotypal and delusional disorders	7/17	41**%**
	F30 – F39 Mood (affective) disorders	9/17	53**%**
	F40 – F49 Neurotic, stress-related and somatoform disorders	1/17	6**%**
Age group	30−40	4/17	24**%**
	40−50	7/17	41**%**
	50−60	5/17	29**%**
	60+	1/17	6**%**
Gender	Male	11/17	65%
	Female	6/17	35**%**
Ethnicity	White British	6/17	35%
	White Other	2/17	12%
	Black/Black British - African	5/17	29%
	Black/Black British - Caribbean	3/17	18%
	Other mixed	1/17	6%
Interview method	Phone call	12/17	71**%**
	Zoom	5/17	29**%**

The themes and subthemes created through framework analysis are summarised in Table 2 and expanded below with quotes for illustration.

**Table 2:** Themes and subthemes.

Theme	Subtheme
Past experiences and expectations of the art forms	Past experiences contributed to participants’ expectations
	It was important for participants to feel capable in their chosen art form
	Participants sometimes had inaccurate expectations of the art forms
Expectations and experiences of social interactions in the groups	Participants expected to share experiences with others in the groups
	Some participants were anxious about the group experience
	Participants had expectations about other group members
Expectations of helpfulness	Participants expected to be able to express themselves non-verbally in their chosen art form
	Participants expected engagement with the art form to give their mind a break
	Participants expected to experience enjoyment and an improvement in mood

### Past experience and expectations of the art forms

#### Past experiences contributed to participants’ expectations

The majority of interviewed participants had attended arts therapies sessions in the past (76 %); this was as an inpatient or in the community. Others had experienced their chosen art form as a hobby, such as being a member of a choir. Almost everyone who reported having previously attended arts therapies sessions chose the same modality as they had experience of (12/13).

“*Yeah I knew I would choose art… because I’m an artist is all I would say*”

(Ppt05 – Art therapy)

Sometimes the participants’ past experiences of arts therapies were different to the ERA groups. This was for a variety of reasons including the groups being more or less structured than expected, a different amount of time spent using the art form, or higher or lower numbers of people attending the group.

“*I was going to a music group… it wasn’t like the one at [ERA], it was a bit different, it was mainly singing… There was more instruments at the ERA one. Not as many people but still a few people*”

(Ppt06 – Music therapy)

Despite some differences, those who had attended arts therapies groups in the past generally had realistic expectations of the ERA groups. Everyone seemed to understand that they would be encouraged to actively engage with the art form. It was not clear whether the video decision-aids contributed to this understanding or whether these expectations were mostly from past experiences.

#### It was important for participants to feel capable in their chosen art form

Sometimes participants were worried about being ‘good enough’ at the art form. Preferences depended on how competent the participants felt in using that art form to enable the therapeutic process. If participants felt they would not be able to use an art form well, it was unlikely to be their preferred modality. There were a number of reasons why participants did not feel confident in using an art form, such as not having an ‘innate’ skill, or having found it difficult at school, or because of physical limitations.

“*I wasn’t keen on dancing. What was the other one, the art? Now I’ve always been rubbish at art*”

(Ppt03 – Music therapy)

“*Yeah, I just chose because the other one was the music therapy but because of my health and my, also my health concerns so I can’t move much, so I couldn’t do with the dancing*”

(Ppt13 – Art therapy)

#### Participants sometimes had inaccurate expectations of the art forms

Participants spoke about what they were expecting to do with the creative arts mediums. There were some mismatched expectations about how much time would be spent talking vs using the art forms in the group. Some participants expressed surprise both that there was more active arts engagement than they expected, or more talking than they had expected.

“*The issue, I felt there wasn’t, often there wasn’t enough time to really get stuck into it… it was good using the art materials but it was difficult to strike a balance between doing the actual art and the talking and checking in in the beginning”*

(Ppt02 – Art therapy)

One participant said he would have liked to have done music if it had been in a recording studio, but upon watching the videos he realised it was music-making with acoustic instruments, suggesting that the videos contributed to a more realistic expectation of the use of the arts mediums. Participants reflected on the use of the art materials, musical instruments and dance-movement props in the sessions and how they were able to explore them, learn new skills and gain confidence. This was sometimes surprising to the participants as they did not expect to be able to engage with the art forms and have these experiences.

*“Well we danced. We had done movement and then we had to pick up cards and you had to explain, we didn’t have to, if you didn’t want to… explain why you picked up a certain card so I found that quite useful… I was quite surprised that I actually participated without feeling, I didn’t really feel embarrassed by anything”*

(Ppt16 – Dance-movement therapy)

### Expectations and experiences of social interactions in the groups

#### Participants expected to share experiences with others in the groups

Participants expected that they would meet others with similar experiences and they would share their stories. This expectation appeared to be met and participants found the groups to be supportive and friendly. There was a sense of belonging and kinship as participants opened up to share their own life stories and experiences of mental distress.

*“EM: What else were you expecting from the art therapy group when you joined the ERA trial?*

*Ppt08: Meet other likeminded people with the same experiences, similar experiences and then just to see how you just grow and nurture during the process of art therapy”*

(Ppt08 – Art therapy)

*“I felt like I belonged, I felt a sense of belonging, that’s the first and the utmost thing.”*

(Ppt09 – Art therapy)

#### Some participants were anxious about the group experience

Some participants felt concerned about the social side and whether they would fit in or be judged by other group members. However, these fears were usually unfounded, and participants were surprised by the acceptance they felt in the groups.

“*Yeah it was totally the opposite of what I have been beating myself up about all the time. Everything that I imagined and practiced and rehearsed in order to be myself and to act normal, that was my theme, to try to act normal like everybody else and it wasn’t even necessary. It wasn’t necessary, I didn’t need to use none of that*”

(Ppt12 – Art therapy)

Conversely, the group experience was challenging for some. One participant found it overwhelming to hear about other group members’ difficult experiences and also disliked being filmed for the research. He found he was feeling anxious in the sessions and then “oversharing” himself, and he decided to drop out of the group. Another participant did not attend any of the groups but remained involved in the trial. She said that she wanted to go but her anxiety levels were too high, and she was not able to.

“*I think I gave into my anxieties, because I hadn’t, for a while I hadn’t accepted that I was dealing with mental health, so that was a challenge in itself. But to then go to the groups, there was always an excuse, so I think I gave in to the fear*”

(Ppt11 – Dance-movement therapy)

#### Participants had expectations about other group members

One participant said he was looking forward to being in a group as his past experience of art therapy had been one-to-one and this had been an intense experience. Some said that they were surprised by the numbers of people in the groups – either that they were smaller than expected or too large. One participant was disappointed when people dropped out of his group, whereas another stopped going to the group when more people joined because he found the music being created was too loud and noisy. It seemed that most participants had an idea of what they thought the group would be like, and how the other group members would behave.

*“…the more people that went to the group, the more uncomfortable I felt. So at first there was only three people and then more people came and I started to feel uncomfortable and then there was the train journey as well, so I stopped, I had to stop going”*

(Ppt06 – Music therapy)

### Expectations of helpfulness

#### Participants expected to be able to express themselves non-verbally in their chosen art form

This expectation was often related to how capable they felt at using that art form, i.e. if they had previous experience and skills in an art form, it would be easier for them to express themselves that way. Participants also spoke about their past experiences of non-verbal expression in the arts therapies as a reason why they held their preference.

“*I think there were so many times I was feeling depressed, or suicidal, in the group at the time and I put them all on paper and I felt ten times better, because they were on paper. So I didn’t take the feelings home with me*.”

(Ppt01 – Art therapy)

“*EM: So, can you sum up what’s been helpful about the therapy for you?*

*Ppt04: Well to be able to sing and express myself. To boost my confidence of being able to do something in a way and I hope when you listen to that, you would feel inspired as well”*

(Ppt04 – Music therapy)

#### Participants expected engagement with the art form to give their mind a break

Using an art form as an escape, or a break from their problems, was an important consideration for the participants’ decision-making. For many, past experiences of being creative and using the arts had offered some respite from their difficult thoughts.

*“…just to be singing out and dance to the rhythm and forget about everything that is happening. That is why I chose music therapy because with music it’s you forget about some of the things*”

(Ppt04 – Music therapy)

*“I think I perhaps might have felt that dance movement would have been a better distraction but I’m not sure… because you’re moving around it’s kind of like people are more focused on the actual movement rather than when you’re sat down it’s easier for people to talk and share how they’re feeling and maybe that’s not entirely helpful all the time”*

(Ppt02 – Art therapy)

#### Participants expected to experience enjoyment and an improvement in mood

Participants expected to enjoy the groups and experience an improvement in mood. They said they wanted to feel more positive and relaxed through using their chosen art form. For some, this expectation came from their past experiences of arts therapies groups or using the arts as a hobby.

“*Because when I was in hospital initially, I was allocated an arts therapist and I really enjoyed it. So, it made my, the first, well one of the first turning points in my breakthrough in recovery, to recovery of mental illness, when I was first introduced to the art therapies team*”

(Ppt09 – Art therapy)

*“I like music, I try and learn the guitar. So, I like music so that’s why I picked it because I like music… As soon as they said that music was an option, I knew straight away yeah… I find music relaxes me, like playing the guitar relaxes me. Occupies my brain for a little while.”*

(Ppt06 – Music therapy)

## Discussion

These interviews offer insight into the experiences of patients who chose an arts therapies group and then attended up to 40 sessions. The results highlight three key areas for patients when making a choice about participating in group arts therapies: past experiences, social interaction and expectations of helpfulness. Almost everyone interviewed had previous experience of the arts therapies (13/17 = 76 %), and all of those who had (except one person) chose an arts modality that they had experience of. This implies that people who attend arts therapies groups are having good experiences. It also suggests that the decision aid videos did not change anyone’s mind, although they may have given participants more realistic expectations. In other research, past therapy experience has been related to more positive expectations about group therapy (MacNair-Semands, 2002). Wider psychotherapy literature suggests that a mismatch in expectations and experiences could lead to non-attendance (Dilgul et al., 2018; Greenberg et al., 2006), although this did not seem to be the case in the current study. Non-attendance appeared to be due to external factors such as personal circumstances, concerns about travelling to the groups or anxiety about the social interaction in the groups, rather than experiences of the art form itself.

Anticipated social interaction in the arts therapies groups appeared to be an important consideration for participants. Social interaction in therapy groups is a key component for positive change (Davies et al., 2015) and group-based arts therapies are designed to promote social interaction amongst members (Carr et al., 2020; Crawford et al., 2012). Although this approach has been found to offer therapeutic benefits (Orfanos et al., 2015), it was a source of anxiety in the current study. Concerns about social interactions have been found to be a barrier to attendance in previous studies of community mental health treatment, and could be related to previous bad experiences of groups (Dilgul et al., 2018), although other studies have suggested that people attending group arts therapies expected to receive peer support from other group members (Pylvänäinen & Lappalainen, 2018). A particular issue for group arts therapies relates to concerns about being ‘good enough’ at the art form. This has been found to cause anxiety in other research into group music therapy (Grocke et al., 2014) and may be more of a problem in groups than in individual arts therapies, due to the potential embarrassment in front of others.

Negative group dynamics have been found to be a reason for non-attendance of community therapy groups (Dilgul et al., 2018) although this did not seem to be a common issue in the current study. Sometimes participants felt frustrated with other group members, or annoyed that the groups were too large or too small, which did contribute to non-attendance. However, most participants described the groups as friendly and supportive. Group cohesion has been suggested to be a crucial element of the therapeutic process (Baker, 2013; Norcross & Wampold, 2011) and group members’ initial perspectives on the group climate have been found to predict change for patients with schizophrenia (Orfanos & Priebe, 2017). This suggests that participants should be guided by their healthcare professionals to consider the group dynamics and their expectations about being part of a group, as part of the shared decision-making process.

The participants expected to be able to use the groups for non-verbal self-expression. Non-verbal, creative expression is an essential component of the arts therapies, and sets them apart from other psychosocial treatment options in mental health services (Fenner et al., 2017; Zubala et al., 2014). Although a number of the participants felt anxious about attending the groups, the art form may have offered a more inviting, and less intimidating, way of connecting with other group members and sharing their experiences.

Many people in the interviews anticipated using the art form as a distraction or to give their mind a rest, and therefore a break from their problems. Some also described how they experienced this in the group and were focused on using the art form. These experiences could also be conceptualised as forms of ‘mindfulness’, a focus on the here and now and increased awareness. These are all well-documented mechanisms of change in the arts therapies (Fidelibus, 2004; Gerber et al., 2018; Peterson, 2015; Rappaport, 2014). Pleasure and enjoyment were also important factors in decision-making and were often mentioned when they spoke about their experiences of the groups. The role of play and pleasure has been highlighted as an key aspect of recovery in psychiatry (Davidson & Roe, 2007) and has been suggested as an important common factor across the arts therapies (Koch, 2017).

### Strengths and limitations

This study was the first to explore patient expectations and experiences of the arts therapies when they were given a choice of arts modality. The involvement of a multi-disciplinary research team, and experts by experience, meant that a wide range of views were taken into consideration throughout the design, analysis and interpretation. There were some limitations in the demographic data collected, including that patient gender was only recorded in a binary manner (male or female). This means that nuances regarding gender would have been missed.

The process was somewhat impacted by the COVID-19 pandemic; the ERA trial was paused in March 2020, limiting the number of participants who had completed the arts therapies groups to a single site. This means that the sample did not cover as wide a range of arts therapy choices, patient characteristics and experiences as was originally planned. For example, more participants were from the art therapy groups than the other modalities, which was representative of the distribution of preferences in the trial where art therapy was the most popular modality. It must also be remembered that dramatherapy was not offered as part of the ERA trial, therefore this study does not include the views of those who would have chosen dramatherapy.

The participants in this study expressed a wide range of experiences and the framework analysis approach made it possible to compare and contrast the interview data (Rabiee, 2004). Conducting the interviews over Zoom or the phone may have made the interviews more accessible for some, although many participants did not have the technology available to use video calling (n = 12/17). The client change interview is designed to be completed collaboratively (Elliott et al., 2001), where both the interviewer and the interviewee are looking at the changes as they are being noted down (see Appendix A). It was possible to do this via a shared screen on Zoom, but it was more challenging over the phone. Some participants seemed to get confused or were unable to allocate ratings to their perceived changes. This issue did not usually affect the information provided about preferences and expectations but may have meant that participants were not able to share their experiences as effectively as during an in-person interview. The first author, who conducted all the interviews, reflected that remote interviews seemed more challenging than interviews in person. It seemed more difficult to establish a rapport, and help participants to feel at ease, as well as maintain focus.

The multiple aims of the interviews, for both this project and wider ERA trial process evaluation meant that a lot needed to be covered and may have limited the depth of discussions about the process of making a choice. Also, due to a complex and innovative trial design, by the time the interviews were conducted, it had been over a year since some of the participants had watched the videos and expressed their preferences. It was difficult for many to remember this initial meeting and explore why they chose their preferred arts therapies modality, and some participants said that they did not remember watching the videos at all. Higher quality data on the initial decision-making process could have been obtained if participants were interviewed about their expressed preferences soon after their baseline assessment, and then again after they had experienced the groups. Despite this, all participants were able to talk about the factors important to them in choosing between therapies, the things which surprised them in the groups and their past experiences of the art forms.

## Conclusion

This study offers insight into the decision-making process for participants who chose and attended arts therapies groups. Although each participant had a unique decision-making process, past experiences of the art forms, social interactions in the groups and expectations of helpfulness were common considerations when making a choice about engagement with the arts therapies. These findings could help clinical teams to address key issues during collaborative decision-making discussions with patients.

## Funding sources

This work was supported by a PhD studentship from East London NHS Foundation Trust. The ERA study is funded by the 10.13039/501100000272National Institute for Health Research (NIHR) Health Technology Assessment programme [grant number 17/29/01]. The views expressed are those of the authors and not necessarily those of the NHS, the NIHR or the Department of Health and Social Care.

## Figures and Tables

**Table:** 

**Materials:**	**Yes or No**
Change interview record	
Encrypted digital audio recorder x 2	

**Please label notes and audio file with the following information:**	
Patient ID	
Date of interview	
Your name	

**Table:** 




**Figure:**
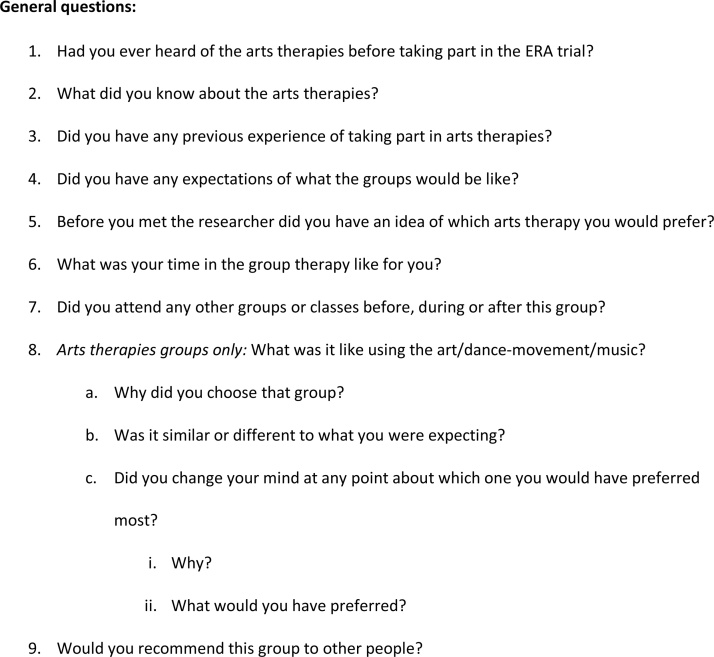


**Figure:**
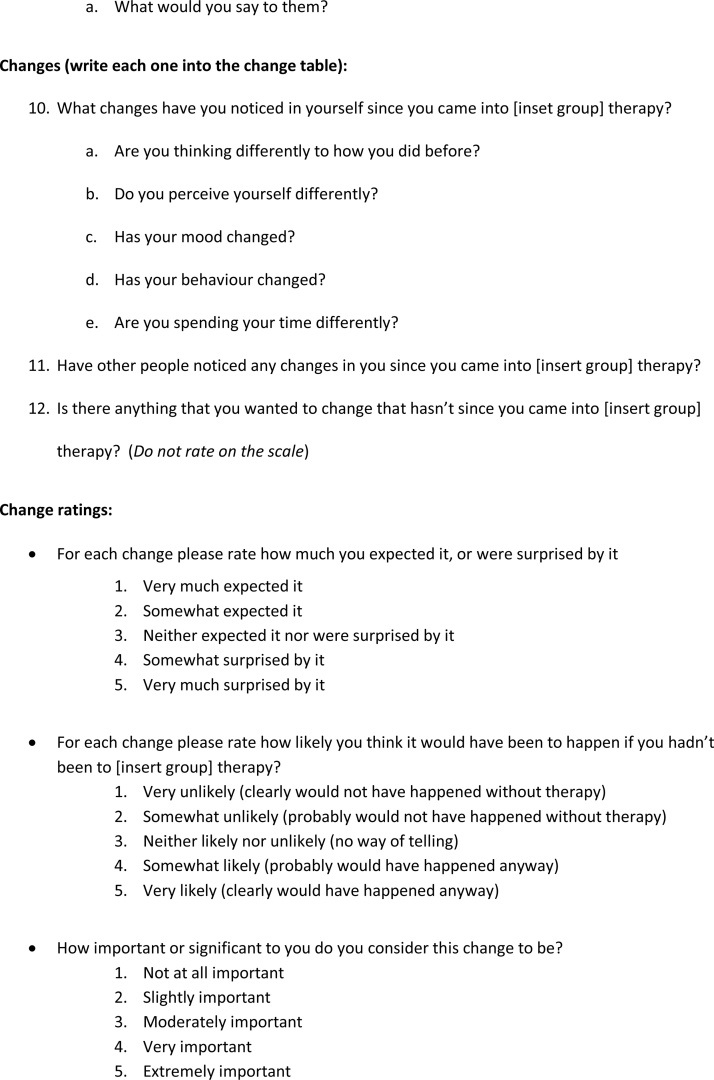


**Figure:**
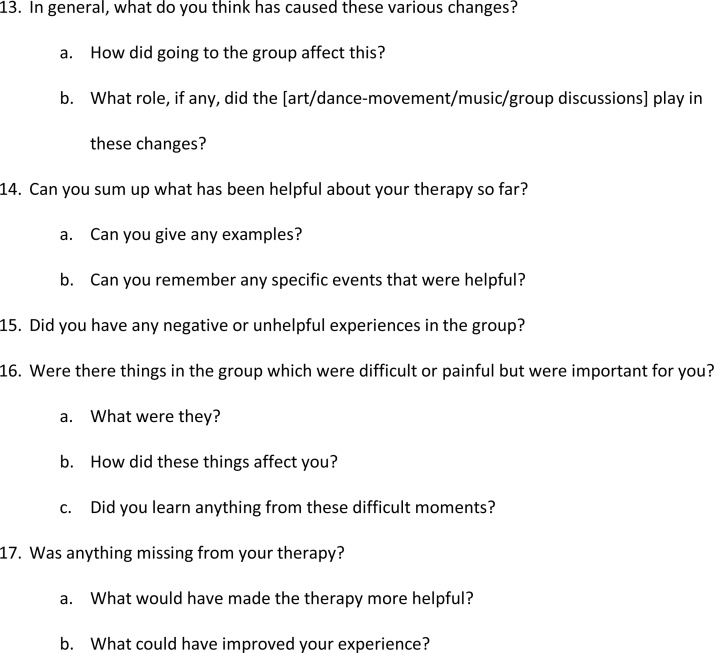


**Table:** 

What was [factor] like for you?	☑	Tell me more about any experiences of [factor]	☑
Meeting others		Whether the group was similar or different to other situations	
Sharing experiences		Being creative or imaginative	
Interacting with others		Playing or being playful	

**Table:** 

Did you have any experiences of [factor]?	☑	Did you notice any changes in [factor]?	☑
Feeling as one/together as a group		Confidence	
Problem solving in the group		Self-esteem	
Learning from other group members		Hope	
Learning about your own strengths and difficulties			
Taking ideas and experiences outside of the group			

## Appendix A ERA End of Therapy Interview incorporating the Client Change Interview

Reference: Elliott, R., Slatick, E., & Urman, M. Qualitative Change Process Research on Psychotherapy: Alternative Strategies. (in press). In J. Frommer and D. Rennie (Eds.), The Methodology of Qualitative Psychotherapy Research. Lengerich: Germany: Pabst Science Publishers.

**Interview strategy**

This interview works best as a relatively unstructured empathic exploration of the client’s experience of therapy. Think of yourself as primarily trying to help the client tell you the story of his or her therapy so far. It is best if you adopt an attitude of curiosity about the topics raised in the interview, using the suggested open-ended questions plus empathic understanding responses to help the client elaborate on his/her experiences. Thus, for each question, start out in a relatively unstructured manner and only impose structure as needed.

Ask client to provide as many details as possible and use the ‘something else’ probe (e.g., “Are there other changes that you have noticed?”): inquire in a non-demanding way until the client runs out of things to say.

As participants mention the factors listed in Q18, tick them off. Factors that have not been mentioned can then be explored.

**Interview Schedule**

**Introduction**:

You have given your consent to take part in an interview about your experiences of group therapy. The interview should take no longer than an hour and will be audio recorded. If you need to stop or take a break at any point, please tell the researcher. What we want to explore are any changes you have noticed since therapy began, what you believe may have brought about these changes, and helpful and unhelpful aspects of the therapy. As you talk about your experiences I will take notes and write down the changes in this table. The main purpose of this interview is to allow you to tell us about the therapy in your own words. This information will help us to further understand how the therapy works; it may help us to improve the therapy in the future. This interview is recorded for later transcription. All of the information you tell us will be kept confidential and your details will not be shared with anyone. The only time we may pass on information about you is if we are concerned about a risk to yourself or someone else. There are no right or wrong answers here; we are interested in your experience so please give us as much detail as you can.

18. *Researcher to tick off factors as they are mentioned during the interview*.

19. Is there anything else you’d like to add about your experience?
